# Credibility of genetic predictors for antiepileptic drug resistance: A systematic Bayesian reappraisal of published meta‐analyses

**DOI:** 10.1002/bcp.70189

**Published:** 2025-08-01

**Authors:** Martina Giacon, Sarah Cargnin, Salvatore Terrazzino

**Affiliations:** ^1^ Department of Pharmaceutical Sciences University of Piemonte Orientale Novara Italy; ^2^ Department of Health Sciences University of Piemonte Orientale Novara Italy

**Keywords:** BFDP, drug response, epilepsy, FPRP, gene polymorphisms, meta‐analyses, Venice criteria

## Abstract

We systematically reappraised meta‐analyses of pharmacogenetic studies to evaluate the credibility of association between gene polymorphisms and resistance to anti‐epileptic drugs (AEDs). A systematic search was performed in PubMed, Web of Knowledge, Cochrane Library and OpenGrey up to April 2025. The methodological quality of the included systematic meta‐analyses was evaluated with the AMSTAR‐2 tool, and the credibility of the genetic comparison results was determined by the Venice criteria and two Bayesian analytic approaches, false positive reporting probability (FPRP), and Bayesian false discovery probability (BFDP). Of the 33 studies identified, 32 were systematic meta‐analyses, all of which were rated as critically low quality by AMSTAR‐2. Our reassessment indicated seven single nucleotide polymorphisms (SNPs) of four genes—ABCB1 (rs1045642, rs2032582), ABCC2 (rs717620, rs3740066), GABRG2 (rs211037) and SCN1A (rs2298771, rs10167228)—which could be regarded as potential determinants of response to AED. Among these, only ABCB1 rs2032582 (G *vs*. A and GG *vs*. GA + AA) was found to be noteworthy in Caucasian epilepsy patients under FPRP or BFDP at the pre‐specified probability level of 0.001. However, the application of the Venice criteria to such relationships identified as weak the strength of the cumulative evidence for epidemiological relationship because of a potential publication bias. Our findings, illustrating the poor yield of genetic predictors from meta‐analyses of candidate gene studies, underscore the need for large‐scale genome‐wide association studies (GWAS) and subsequent replication studies for identification of robust predictors of resistance to AEDs.

## 1 INTRODUCTION

Epilepsy, a prevalent neurological disease that affects about 50 million individuals around the world, is characterized by recurrent and unprovoked seizures.^1^ Antiepileptic drugs (AEDs) are the mainstay of therapy, and goals include seizure control and improvement in quality of life. Nevertheless, it is difficult to obtain adequate control of seizures as evidenced by the 20–30% of patients who do not respond to existing treatment.^2^, ^3^ This major limitation to optimal medical care highlights the pressing requirement to elucidate determinants of treatment variability and to realize personalized treatment approaches. The reasons for variability in AED response involve a complicated relationship between factors including patient factors (age, sex, co‐morbidities), environmental factors and genetic predisposition.^4^ Among these, genetic profile has been recognized as key determinant of both drug efficacy and tolerability.^5^ Thus, the discovery of the specific genetic polymorphisms that affect individual AED responses is key to taking precision medicine forward to make it possible to tailor treatment in such a way as to optimize drug efficacy and tolerability.^6^

In the last decade, considerable research effort, including many candidate gene association studies, have attempted to pinpoint genetic determinants of AED resistance.^7^, ^8^, ^9^ Such initiatives resulted in several meta‐analyses combining available evidence on associations of individual variations (i.e., polymorphisms) with treatment response. Although meta‐analyses may be a way of enhancing statistical power and delivering more robust effect estimates, their standard approaches often depend heavily on the fixed *P*‐value thresholds, which may result in an increased risk of type I errors (false positives) and in some cases may compromise the trustworthiness of combined results.^10^ Systematic review of evidence is therefore important to separate true genetic associations from false positive findings. These evaluations apply a range of statistical methodological approaches to judge the epidemiological or biological plausibility of reported associations. Although previous meta‐analyses have indicated associations between some gene polymorphisms and response to AED, an overall and systematic evaluation of the credibility of these associations is not available.

In order to bridge this important gap, here, we systematically reconsidered and re‐analysed all of the available meta‐analytic data related to the association between gene polymorphisms and resistance to AED. Our systematic review used the Venice criteria, a well‐known framework for evaluation of the epidemiologic evidence with regard to strength, consistency and absence of bias.^11^ In addition, we assessed the statistical significance of positive findings using two Bayesian methods: the false positive report probability (FPRP)^12^ and the Bayesian false discovery probability (BFDP),^13^ which provide probability estimates of the chance of observing a statistically significant finding if there is no true association.

## 2 METHODS

### 2.1 Literature search and selection criteria

The systematic review was conducted in accordance with the PRISMA guidelines^14^ and its protocol was prospectively registered in INPLASY (registration number 2024120072). Two reviewers (M.G. and S.T.) systematically searched PubMed, Web of Knowledge, Cochrane Library and OpenGrey databases up to April 11, 2025. The search strategy included a combination of the index terms: (polymorphism OR polymorphisms OR SNP OR SNPs OR genetic OR genetics OR variant OR variants) AND meta‐analysis AND (drug OR drugs OR response OR responsiveness OR resistance) AND (epilepsy OR epileptic OR seizure). The eligible studies were meta‐analyses of observational studies on genetic variation and antiepileptic treatment response in patients with epilepsy, and measured the response to antiepileptic drugs as a dichotomous outcome. Eligibility criteria were that studies provided OR and 95% CIs, which were used to calculate FPRP^12^ and Bayesian false discovery probability (BFDP).^13^ Studies of non‐human, narrative reviews, meta‐analyses that were not about the research topic, genome‐wide association studies (GWAS), case reports, conference abstracts and editorials were excluded. To obtain full identification, reviewers screened abstracts and titles, full text and manually screened the references of the included studies. Language restrictions were not imposed. Any discrepancies between the investigators were resolved through discussion until a consensus was reached.

### 2.2 Data extraction

Full data extraction was carried out of all included studies. These were primary study identifiers (first author, year of publication and location), search information (database and date) and key population descriptors (participant ethnicity, antiepileptic drug studied). We collected the following information for each gene polymorphism analysed in the meta‐analysis: number of studies or cohorts included; total sample size; the name of the polymorphism; the genetic model analysed (allele, dominant or recessive), and the corresponding odds ratio (OR), 95% confidence interval (CI) and *P‐*value. We also extracted from the original meta‐analysis: the model used (fixed *vs*. random) and measures of heterogeneity (index of inconsistency [*I* ^2^], Q statistic *P*‐value) and the *P*‐value from the Egger test for publication bias. If the *P*‐values for the pooled effect, the evaluation of heterogeneity or the Egger test were not provided but a statistically significant combined effect was presented in a meta‐analysis, these missing *P*‐values were approximated from the forest plot of the main references (if available) with the help of the metafor package (version 4.8‐0) in RStudio (version 4.4.3). The corresponding author of the article was sent an email if the forest plot was not available in the article. Similar subgroup results based on antiepileptic drug type, patient ethnic origin and paediatric status were also included. Any disagreements between the reviewers (M.G., S.T.) were resolved through discussion and consensus.

### 2.3 Study quality and cumulative evidence assessment

The first step was to evaluate the methodological quality of the included systematic reviews based on the AMSTAR‐2 tool, which contains 16 items about confidence in the findings of the review.^15^ We then used the Venice criteria^11^ to assess the plausibility of the positive associations we identified. Overall credibility of the evidence was rated according to the Venice criteria (strong, moderate or weak) on the three dimensions, including amount of evidence, consistency of effect, and risk of bias. In particular, the amount of evidence was classified by total sample size (A: >1000; B: 100–1000; C: ≤100). Heterogeneity was considered to measure the replicate reliability through the *I* ^2^ statistic (A: <25%; B: 25–50%; C: ≥50%). Risk of bias was classified as Grade A if the *P*‐value of the Egger test was ≥0.05, and bias likelihood was low; Grade B if the *P*‐value was also >0.05, but potential concerns in bias were observed; and Grade C if the *P*‐value was < 0.05, or low likelihood OR was ≤0.87 or ≥1.15. A “High” overall credibility required Grade A in all domains, “Moderate” permitted Grades A and B, while “Low” was assigned if any domain received a Grade C.

### 2.4 Statistical analysis

For Bayesian analysis, if there were several studies that reported significant results of the same polymorphism variant, the largest study was considered, unless the results regard different subgroups (e.g., ethnic group, antiepileptic drug type or paediatric status). We used the false positive report probability (FPRP) and Bayesian false discovery probability (BFDP) to assess the importance of the significant genetic associations. FPRP calculates the probability of a true positive association conditional upon an observed significant finding,^12^ considering the magnitude of the observed *P*‐value, statistical power and prior probability of an association. For FPRP, calculations were conducted using two prior probability thresholds representing the average expectation for the candidate genes (0.05 [medium] and 0.001 [low]). Statistical power was computed for ORs of 1.50 (0.67 the reciprocal) and 2.0 (0.50 the reciprocal). On the other hand, BFDP also offers a computationally simple evaluation, but the importance threshold is based on the trade‐off between the relative costs of false discovery and false non‐discovery.^13^ In contrast to FPRP, BFDP is not related to statistical power and based on a logistic regression estimator. We performed the calculations using the prior probabilities 0.05 and 0.001, and we used a noteworthiness threshold of <0.8, which corresponds to a scenario where a false non‐discovery is four times more costly than a false discovery. A statistically significant genetic association was considered noteworthy if its FPRP was < 0.2 or if its BFDP was < 0.8. The calculations were made using the Excel spreadsheets of Wacholder et al. ^12^ and Wakefield.^13^

### 2.5 Assessment of biological plausibility via eQTL analysis

To examine the potential functional importance of the statistically significant polymorphisms detected in the primary meta‐analyses presented in this review, we accessed publicly available expression quantitative trait loci (eQTL) databases. Specifically, data were interrogated with tools such as the Genotype‐Tissue Expression (GTEx) portal^16^ to assess whether important variants being studied could result in different expression levels of their corresponding genes across pertinent brain tissue in humans. This search was conducted in an illustrative way to evaluate the biological relevance of genetic associations found in the literature and to search for potential regulatory effects of these variants in the target organ itself. Results from this eQTL analysis informed the discussion about putative mechanisms, but were not included in the formal process of scoring credibility (Venice criteria, FPRP, BFDP) of the meta‐analysed associations themselves.

## 3 RESULTS

### 3.1 Literature screening and meta‐analysis characteristics

**FIGURE 1:**
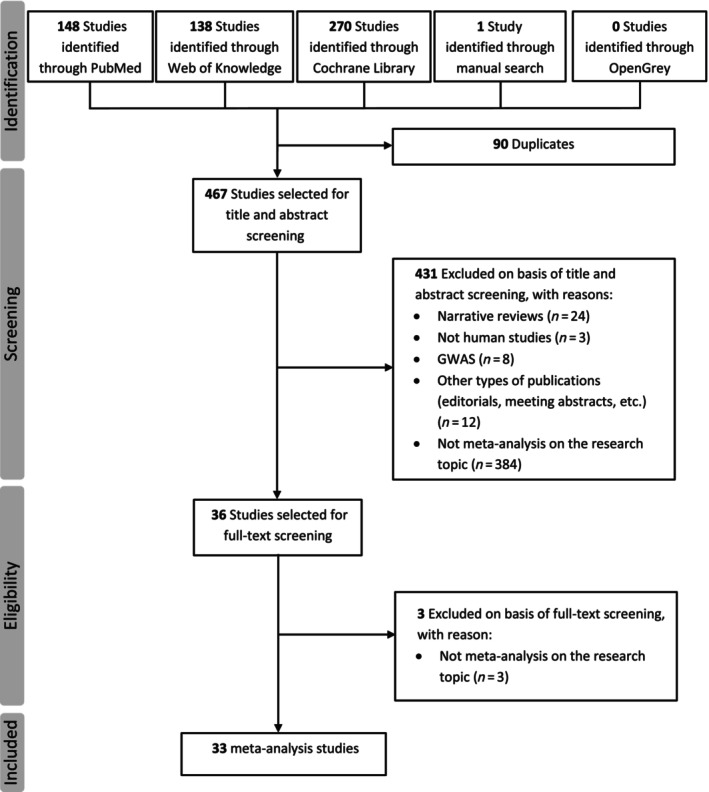
PRISMA flow diagram illustrating the study selection process. The diagram illustrates the process for filtering eligible studies by database or manual searching and removing duplicate studies, as well as the selection of titles and abstracts at the initial screening and the full text at the later eligibility assessment, and the number of meta‐analyses included in the systematic reappraisal of associations of gene polymorphisms with antiepileptic drug response. GWAS, genome‐wide association study.

Initially, we identified 557 publications through literature search in PubMed, Web of Knowledge, Cochrane Library and OpenGrey. Following deduplication (n = 90), we screened the title, abstract, and then the full text of the 467 records. This step resulted in the exclusion of 431 studies. Finally, 33 meta‐analyses on the relationship between gene polymorphism and resistance to AEDs were eligible and included in this systematic review.^17^, ^18^, ^19^, ^20^, ^21^, ^22^, ^23^, ^24^, ^25^, ^26^, ^27^, ^28^, ^29^, ^30^, ^31^, ^32^, ^33^, ^34^, ^35^, ^36^, ^37^, ^38^, ^39^, ^40^, ^41^, ^42^, ^43^, ^44^, ^45^, ^46^, ^47^, ^48^, ^49^ A flowchart in Figure 1 shows the detailed literature selection process. The 33 meta‐analyses included in the systematic review were published between 2007 and 2025, and presented evidence of geographical distribution, with 23 (69.7%) from China. Methodologically, 24 (72.7%) employed a minimum of four databases in their literature searches and 29 (87.9%) were published within a year of their reported search dates. Three meta‐analyses^23^, ^33^, ^36^ were limited to paediatric epileptic patients, whereas the others did not have age inclusion criteria. Overall, 29 single nucleotide polymorphisms (SNPs) from 12 genes were considered in the pooled single locus analysis: ABCB1 (rs1128503, rs2032582, rs1045642), ABCC2 (rs717620, rs3740070, rs3740066, rs2273697, −1774delG), ABCG2 (rs2231142, rs2231137), CYP3A4 (rs2242480), CYP3A5 (rs776746), EPHX1 (rs2234922, rs1051740), GABRA1 (rs2279020), GABRA6 (rs3219151), GABRG2 (rs211037), SCN1A (rs6722462, rs6432860, rs4667866, rs3812718, rs2298771, rs1461197, rs1020853, rs10188577, rs10167228), SCN2A (rs2304016, rs17183814) and SLC6A11 (rs2304725). Moreover, three SNPs of the ABCB1 gene (rs1128503, rs2032582, rs1045642) and three SNPs of ABCC2 (rs717620, rs2273697, rs3740066) were evaluated in haplotype combination. The gene polymorphisms included in each meta‐analysis are described in Table 1, together with additional main features from these studies.

**TABLE 1:** Characteristics of included meta‐analyses on genetic variants and antiepileptic drug resistance.

Study	Country	Search database	Search date	Drug	SNP investigated (number of included studies)
Leschziner 2007^17^	United Kindgom	None	None	AEDs	ABCB1 rs1045642 (*n* = 3)
Bournissen 2009^18^	Canada	Medline, Embase, Scopus, WOS	September 2007	AEDs	ABCB1 rs1045642 (*n* = 11)
Haerian 2010^19^	Malaysia	Cochrane Library, Medline, Embase, Google	February 2010	AEDs	ABCB1 rs1045642 (*n* = 24)
Haerian 2011^20^	Malaysia	Medline, Embase, Cochrane	August 2010	AEDs	ABCB1 rs1045642 (*n* = 25) ABCB1 rs2032582 (*n* = 12) ABCB1 rs1128503 (*n* = 10) ABCB1 haplotype (*n* = 6)
Haerian 2013^21^	Malaysia	Medline, Embase, Cochrane	February 2013	AEDs	SCN1A rs3812718 (*n* = 8) SCN1A rs2298771 (*n* = 6) SCN2A rs17183814 (*n* = 5)
Grover 2013^22^	India	Embase, Medline, WOS, Cochrane	8 September 2012	AEDs	ABCC2 rs717620 (*n* = 6) ABCC2 rs2273697 (*n* = 8) ABCC2 rs3740066 (*n* = 6)
Sun 2014^23^	China	PubMed, Medline, Embase, CNKI	September 2013	AEDs	ABCB1 rs1045642 (*n* = 8)
Lv 2014^24^	China	CNKI, CBM, WanFang, MedCH international medical abstract, PubMed, ScienceDirect, other	31 March 2014	AEDs	ABCB1 rs1045642 (*n* = 25)
Li 2014^25^	China	PubMed, Embase, WanFang, CNKI, Chongqing VIP	February 2013	AEDs	ABCB1 rs1045642 (*n* = 35 articles including 38 independent case–control studies)
Cheng 2014^26^	China	Cochrane Library, Medline, Embase, CBM disc, CNKI, VIP, WanFang	August 2013	AEDs	ABCB1 rs1045642 (*n* = 14)
Chen 2014^27^	China	PubMed, Embase	31 March 2013	AEDs	ABCC2 rs2273697 (*n* = 6)
Wang 2015^28^	China	Pubmed, WOS, Cochrane, Embase	May 2014	AEDs	ABCC2 rs2273697 (*n* = 8) ABCC2 rs717620 (*n* = 5) ABCC2 rs3740066 (*n* = 6) ABCC2 rs3740070 (*n* = 2) ABCC2 − 1774G > delG (*n* = 3) ABCC2 haplotype
Yu 2015^29^	China	PubMed, Embase, Google Scholar, CNKI	September 2014	AEDs	ABCB1 rs2032582 (*n* = 15)
Li 2015^30^	China	Cochrane Library, Medline, Embase, PubMed, ScienceDirect, CNKI, CBM, MedCH international medical abstract, WanFang	October 2014	AEDs	ABCB1 rs1045642 (*n* = 28 articles including 30 independent case–control studies)
Li 2015^31^	China	Pubmed, Embase, WOS, CNKI, Chinese Biomedicine	15 July 2014	AEDs	ABCB1 rs1045642 (*n* = 53) ABCB1 rs1128503 (*n* = 20) ABCB1 rs2032582 (*n* = 25) ABCB1 haplotype (*n* = 10)
Qian 2017^32^	China	Embase, PubMed, Cochrane Library, CNKI	May 2016	AEDs	ABCC2 rs2273697 (*n* = 13) ABCC2 rs3740066 (*n* = 8) ABCC2 rs717620 (*n* = 10) ABCC2 haplotype
Lv 2017^33^	China	PubMed, Medline, Embase, CNKI	October 2016	AEDs	ABCB1 rs1045642 (*n* = 11)
Couchi 2017^34^	Tunisia	Pubmed, Cochrane Library	November 2016	AEDs	ABCB1 rs1045642 (*n* = 13)
Bao 2018^35^	China	PubMed, Embase, Cochrane Library, Medline	October 2017	AEDs	SCN1A rs2298771 (*n* = 4) SCN1A rs3812718 (*n* = 8)
Wang 2018^36^	China	PubMed, Embase, WOS, CNKI, WanFang	20 May 2018	VPA	SCN1A rs3812718 (*n* = 13)
Zhao 2019^37^	China	PubMed, Embase, Cochrane library, CNKI, Chinese Science and Technique Journals, CBM disc, WanFang	September 2019	CBZ	EPHX1 rs1051740 (*n* = 4) EPHX1 rs2234922 (*n* = 4)
Zhang 2021^38^	China	PubMed, Embase, WOS	August 2020	CBZ	SCN1A rs3812718 (*n* = 6) SCN1A rs2298771 (*n* = 4)
Fan 2021^39^	China	PubMed, Embase, Cochrane library, CNKI, Chinese Science and Technique Journals, WanFang	5 July 2021	CBZ	ABCB1rs1045642 (*n* = 8) ABCB1 rs2032582 (*n* = 4) ABCB1 rs1128503 (*n* = 2)
Zan 2021^40^	China	PubMed, EBSCO, Ovid, CNKI	October 2020	AEDs	ABCC2 rs2273697 (*n* = 16) ABCC2 rs717620 (*n* = 12) ABCC2 rs3740066 (*n* = 10) ABCG2 rs2231137 (*n* = 4) ABCG2 rs2231142 (*n* = 4)
Yang 2021^41^	China	PubMed, Medline, CNKI	2 May 2020	AEDs	SCN2A rs2304016 (*n* = 4) SCN2A rs17183814 (*n* = 6)
Li 2021^42^	China	PubMed, Embase, Cochrane Library, WanFang, CNKI	June 2020	AEDs	SCN1A rs2298771 (*n* = 13) SCN1A rs10188577 (*n* = 6) SCN2A rs17183814 (*n* = 9) SCN2A rs2304016 (*n* = 7)
Zhao 2021^43^	China	PubMed, Embase, Cochrane library, CNKI, Chinese Science and Technique Journals, CBM disc and WanFang	January 2021	CBZ	CYP3A4 rs2242480 (*n* = 1) CYP3A5 rs776746 (*n* = 1) SCN1A rs3812718 (*n* = 5) SCN1A rs2298771 (*n* = 3)
Chen 2022^44^	China	PubMed, Medline, Embase, WOS, Cochrane Library, CNKI	June 2020	AEDs	ABCB1 rs1045642 (*n* = 62 articles including 70 independent case–control studies)
Zhang 2022^45^	Brazil	Pubmed, Ovid, WOS, CNKI	18 May 2022	AEDs	GABRA1 rs2279020 (*n* = 11) GABRA6 rs3219151 (*n* = 5)
Krami 2022^46^	Morocco	PubMed, Scopus	March 2020	AEDs	ABCB1 rs2032582 (*n* = 33)
Hu 2023^47^	China	PubMed, Embase, Cochrane Library, WOS, Google Scholar, Wanfang, CNKI, VIP	18 April 2023	AEDs	SLC6A11 rs2304725 (*n* = 4) GABRG2 rs211037 (*n* = 8)
Wang 2023^48^	China	Embase, Medline, WOS, CNKI, WanFang	9 April 2023	AEDs	ABCC2 rs717620 (*n* = 18)
Mohammadi 2025^49^	Iran	Pubmed, Scopus, Web of Science	8 November 2024	AEDs	SCN1A rs2298771 (*n* = 25) SCN1A rs3812718 (*n* = 25) SCN1A rs10188577 (*n* = 10) SCN1A rs1020853 (*n* = 2) SCN1A rs1461197 (*n* = 2) SCN1A rs10167228 (*n* = 2) SCN1A rs1972445 (*n* = 2)

Abbreviations: AED, antiepileptic drug; CBM, China Biology Medicine; CBZ, carbamazepine; CNKI, China National Knowledge Infrastructure; NS, not specified; VIP, China Science and Technology Journal; VPA, valproic acid; WOS, Web of Science.

### 3.2 Methodological quality of systematic reviews

Among the 33 identified studies, 32 studies were systematic meta‐analyses (SRs). The only exception was one meta‐analysis without a systematic review part.^17^ When the AMSTAR‐2 measure was used on the 32 systematic meta‐analyses, the quality of all of them was critically low (100%). The performance of the studies was excellent in six domains (100% compliance), namely #1 (PICO components), #3 (justification of the study design), #5 (duplicate study selection), #6 (duplicate data extraction), #8 (detailed description of the study) and #11 (statistical methods). At the other end of the range were three critical areas, including domain #2 (reporting an explicit protocol statement), where 96.9% failed, as well as domain #9 (assessing risk of bias) and domain #13 (considering risk of bias in interpretation). Detailed item scores and AMSTAR‐2 overall quality scores for these reviews can be found in Table 2.

**TABLE 2:** Methodological quality of included systematic meta‐analyses according to AMSTAR2.

Study	Q1	Q2	Q3	Q4	Q5	Q6	Q7	Q8	Q9	Q10	Q11	Q12	Q13	Q14	Q15	Q16	Overall quality
Bournissen 2009^18^	Y	N	Y	PY	Y	Y	PY	Y	N	N	Y	N	PY	Y	PY	Y	Critically low
Haerian 2010^19^	Y	N	Y	PY	Y	Y	N	Y	N	N	Y	N	N	Y	PY	N	Critically low
Haerian 2011^20^	Y	N	Y	PY	Y	Y	N	Y	N	N	Y	N	N	Y	PY	Y	Critically low
Haerian 2013^21^	Y	N	Y	PY	Y	Y	N	Y	N	N	Y	N	N	Y	PY	Y	Critically low
Grover 2013^22^	Y	N	Y	PY	Y	Y	Y	Y	N	N	Y	N	N	Y	Y	Y	Critically low
Sun 2014^23^	Y	N	Y	PY	Y	Y	N	Y	N	N	Y	N	N	Y	Y	N	Critically low
Lv 2014^24^	Y	N	Y	PY	Y	Y	N	Y	N	N	Y	N	N	Y	Y	Y	Critically low
Li 2014^25^	Y	N	Y	PY	Y	Y	N	Y	N	N	Y	N	N	Y	Y	Y	Critically low
Cheng 2014^26^	Y	N	Y	PY	Y	Y	N	Y	N	N	Y	N	N	Y	Y	N	Critically low
Chen 2014^27^	Y	N	Y	PY	Y	Y	Y	Y	N	N	Y	N	N	Y	Y	Y	Critically low
Wang 2015^28^	Y	N	Y	PY	Y	Y	N	Y	N	N	Y	N	N	Y	Y	N	Critically low
Yu 2015^29^	Y	N	Y	PY	Y	Y	Y	Y	N	N	Y	N	N	Y	Y	Y	Critically low
Li 2015^30^	Y	N	Y	PY	Y	Y	Y	Y	N	N	Y	N	N	Y	Y	N	Critically low
Li 2015^31^	Y	N	Y	PY	Y	Y	N	Y	N	N	Y	N	N	Y	Y	Y	Critically low
Qian 2017^32^	Y	N	Y	PY	Y	Y	Y	Y	N	Y	Y	N	N	Y	Y	Y	Critically low
Lv 2017^33^	Y	N	Y	PY	Y	Y	N	Y	N	N	Y	N	N	Y	Y	Y	Critically low
Couchi 2017^34^	Y	N	Y	PY	Y	Y	Y	Y	N	Y	Y	N	N	Y	Y	Y	Critically low
Bao 2018^35^	Y	N	Y	PY	Y	Y	Y	Y	N	Y	Y	N	N	Y	Y	Y	Critically low
Wang 2018^36^	Y	N	Y	PY	Y	Y	Y	Y	N	N	Y	N	N	Y	Y	Y	Critically low
Zhao 2019^37^	Y	N	Y	PY	Y	Y	Y	Y	N	Y	Y	N	N	Y	Y	Y	Critically low
Zhang 2021^38^	Y	N	Y	PY	Y	Y	Y	Y	N	N	Y	N	N	Y	Y	Y	Critically low
Fan 2021^39^	Y	N	Y	PY	Y	Y	Y	Y	N	N	Y	N	N	Y	N	Y	Critically low
Zan 2021^40^	Y	N	Y	PY	Y	Y	N	Y	N	N	Y	N	N	Y	Y	Y	Critically low
Yang 2021^41^	Y	N	Y	PY	Y	Y	N	Y	N	N	Y	N	N	Y	Y	Y	Critically low
Li 2021^42^	Y	N	Y	PY	Y	Y	Y	Y	N	Y	Y	N	N	Y	Y	Y	Critically low
Zhao 2021^43^	Y	N	Y	PY	Y	Y	Y	Y	N	Y	Y	N	N	Y	Y	Y	Critically low
Chen 2022^44^	Y	N	Y	PY	Y	Y	Y	Y	N	Y	Y	N	N	Y	Y	Y	Critically low
Zhang 2022^45^	Y	N	Y	PY	Y	Y	Y	Y	N	Y	Y	N	N	Y	Y	Y	Critically low
Krami 2022^46^	Y	N	Y	PY	Y	Y	Y	Y	N	Y	Y	N	N	Y	Y	Y	Critically low
Hu 2023^47^	Y	N	Y	PY	Y	Y	Y	Y	Y	N	Y	Y	N	N	Y	Y	Critically low
Wang 2023^48^	Y	N	Y	PY	Y	Y	Y	Y	N	Y	Y	N	N	Y	Y	Y	Critically low
Mohammadi 2025^49^	Y	PY	Y	Y	Y	Y	N	Y	N	N	Y	PY	N	Y	Y	Y	Critically low

**Q1:** Were patient/problem, intervention, comparison and outcome (PICO) components taken into consideration when drawing up the research questions and inclusion criteria? **Q2:** Does the review report explicitly state that the methods of the review were decided before commencing the review, and if these methods were changed does the report justify the change? **Q3:** Have the authors provided an explanation for their choice of the study designs they include in the review? **Q4:** Was the authors' strategy for literature search sufficiently comprehensive? **Q5:** Were any duplicates of studies selected? **Q6:** Were any duplicate data extracted? **Q7:** Were excluded studies listed and a justification given for the exclusion? **Q8:** Is the detail with which the authors describe the studies adequate to the task? **Q9:** Has the risk of bias in individual studies been addressed satisfactorily through an appropriate technique? **Q10:** Are the individual studies' funding sources reported in the review? **Q11:** Have the authors of the review employed appropriate methods to statistically combine the results of any meta‐analysis conducted? **Q12:** Have the review authors assessed what impact risk of bias in individual studies might have on the results of their meta‐analyses or on other evidence formation? **Q13:** Is risk of bias in the individual studies adequately addressed by the authors in the discussion and interpretation of their review's results? **Q14:** Where heterogeneity was observed in the review results, have the authors attempted a satisfactory discussion and explanation thereof? **Q15:** If quantitative synthesis was performed, have the authors adequately addressed the issue of publication bias (small study bias) and offered a discussion of how it might be likely to impact the review results? **Q16:** Have the review authors reported on any conflicts of interest, including through funding of their review, which might arise?

AMSTAR2, Assessment of Multiple Systematic Reviews 2; N, no: negative response or response not available; PY, partial yes: incomplete adherence to the criteria; Y, yes: positive response.

AMSTAR 2 Critical domains: Protocol registered before commencement of the review (item 2); Adequacy of the literature search (item 4); Justification of excluding individual studies (item 7); Risk of bias from individual studies being included in the review (item 9); Appropriateness of meta‐analytical methods (item 11); Consideration of risk of bias when interpreting the results of the review (item 13); Assessment of presence and likely impact of publication bias (item 15).

### 3.3 Bayesian re‐analysis of meta‐analyses

Overall, we found 23 significant genetic comparisons of seven SNPs in four genes: ABCB1 (rs1045642 and rs2032582), ABCC2 (rs717620 and rs3740066), GABRG2 (rs211037) and SCN1A (rs2298771, rs10167228)) in the meta‐analyses included in the systematic review (Table 3). Among them, only that of ABCB1 rs2032582 in the Caucasian patients was still significant under FPRP or BFDP with a prespecified probability level of 0.001 (G *vs*. A and GG *vs*. GA + AA). However, the credibility of these findings based on cumulated epidemiologic evidence by Venice criteria in these two genetic contrasts in Caucasian patients was considered to be weak as a result of potential publication bias (Egger's *P* < 0.05). In addition, seven comparisons under FPRP (at statistical powers of both 1.5 and 2.0) or BDFP were considered as noteworthy at the less stringent prior probability of 0.05: the C *vs*. T, the CC *vs*. CT + TT and the CC + CT *vs*. TT models for ABCB1 rs1045642 in Caucasians; and the TT vs CT + CC comparison of ABCC2 rs717620 and the CC *vs*. CT + TT of ABCC2 rs3740066 in the overall analysis and in the subgroup of Asian patients. Among these comparisons, four were moderate and three were weak based on the Venice criteria. Meanwhile, the association of ABCB1 rs2032582 (T + A *vs*. G) in the Asian epilepsia subgroup and the A *vs*. G and AA *vs*. AG + GG models of SCN1A rs2298771 in the overall analysis were rated as strong according to the Venice criteria. Similarly, for the carbamazepine‐treated Asian patients, the GG *vs*. AA + GA comparison of rs2298771 was graded as moderate according to the Venice criteria. Nevertheless, none of these comparisons were found to be noteworthy with either FPRP or BDFP. Results of the non‐significant genetic contrasts of the other 22 SNPs and haplotypes in ABCB1 and ABCC2 are presented in Supplementary Table S1.

**TABLE 3:** Summary of significant (*P* < 0.05) genetic associations with antiepileptic drug (AED) resistance from included meta‐analyses.

Study	Drug	Gene variant	Total subjects or alleles	Comparison	Ethnicity	Model	OR (95% CI)	*P*‐value (estimated)	*P*_het_ (estimated)	*I* ^2^ (%)	Egger *P*‐value (estimated)	FPRP values at prior probability				BFDP 0.05	BFDP 0.001	Venice Criteria	Venice Criteria Score
												OR 1.5		OR 2.0					
												0.05	0.001	0.05	0.001				
Chen 2022^44^	AEDs	ABCB1 rs1045642	29 116	C *vs*. T	Overall	R	1.13 (1.02–1.25)	0.02	0.0001	77	0.838	0.251	0.946	0.251	0.946	0.847	0.997	ACC	W
Chen 2022^44^	AEDs	ABCB1 rs1045642	14 562	CC *vs*. CT + TT	Overall	R	1.20 (1.03–1.39)	0.02	0.00001	71	0.348	0.223	0.938	0.222	0.938	0.804	0.995	ACA	W
Chen 2022^44^	AEDs	ABCB1 rs1045642	11 230	C *vs*. T	Caucasian	R	1.24 (1.09–1.43)	0.002	0.00001	64	(0.164)	**0.056**	0.757	**0.056**	0.756	**0.539**	0.984	ACA	W
Chen 2022^44^	AEDs	ABCB1 rs1045642	2927	CC *vs*. CT + TT	Caucasian	R	1.35 (1.11–1.66)	0.003	0.001	52	(0.364)	**0.091**	0.840	**0.078**	0.816	**0.627**	0.989	ACA	W
Chen 2022^44^	AEDs	ABCB1 rs1045642	2927	CC + CT *vs*. TT	Caucasian	F	1.26 (1.06–1.49)	0.008	0.01	43	(0.061)	**0.118**	0.876	**0.116**	0.873	**0.688**	0.991	ABB	M
Chen 2022^44^	AEDs	ABCB1 rs1045642	506	C *vs*. T	Tunisian	R	0.31 (0.15–0.65)	0.002	0.07	70	NC^a^	0.633	0.989	0.263	0.949	0.877	0.997	BC‐	N/A
Chen 2022^44^	AEDs	ABCB1 rs1045642	253	CC *vs*. CT + TT	Tunisian	F	0.34 (0.20–0.60)	0.0001	0.73	0	NC^a^	0.271	0.951	**0.039**	0.682	**0.692**	0.992	BA‐	N/A
Chen 2022^44^	AEDs	ABCB1 rs1045642	253	CC + CT *vs*. TT	Tunisian	R	0.18 (0.04–0.78)	0.02	0.07	71	NC^a^	0.912	0.998	0.829	0.996	0.942	0.999	BC‐	N/A
Krami 2022^46^	AEDs	ABCB1 rs2032582	882	GG + GA *vs*. AA	Overall	F	0.56 (0.34–0.93)	0.02	0.85	0	0.126	0.656	0.990	0.416	0.974	0.901	0.998	BBA	M
Krami 2022^46^	AEDs	ABCB1 rs2032582	1360	G *vs*. A	Caucasian	F	0.45 (0.34–0.60)	<0.0001	(0.366)	5.4	(0.045)	**0.000**	**0.014**	**0.000**	**0.000**	**0.002**	**0.084**	AAC	W
Krami 2022^46^	AEDs	ABCB1 rs2032582	453	GG *vs*. GA + AA	Caucasian	F	0.24 (0.15–0.38)	<0.0001	(0.130)	46.9	0.041	**0.003**	**0.149**	**0.000**	**0.001**	**0.008**	**0.286**	BBC	W
Yu 2015^29^	AEDs	ABCB1 rs2032582	2547	T + A *vs*. G	Asian	F	1.12 (1.01–1.25)	0.039	0.566	0	(0.9285)	0.450	0.977	0.450	0.977	0.915	0.998	AAA	S
Wang 2023^48^	AEDs	ABCC2 rs717620	4510	TT *vs*. CT + CC	Overall	F	1.68 (1.27–2.21)	(0.0002)	0.418	3.1	0.05	**0.019**	0.499	**0.004**	0.189	**0.232**	0.941	AAC	W
Wang 2023^48^	AEDs	ABCC2 rs717620	2984	TT *vs*. CT + CC	Asian	F	1.70 (1.26–2.29)	(0.0005)	0.15	29.3	(0.09)	**0.043**	0.701	**0.011**	0.359	**0.380**	0.970	ABA	M
Zan 2021^40^	AEDs	ABCC2 rs3740066	3341	CC *vs*. CT + TT	Overall	R	2.29 (1.44–3.64)	0.0005	0.05	47.1	0.804	**0.191**	0.926	**0.030**	0.617	**0.638**	0.989	ABA	M
Zan 2021^40^	AEDs	ABCC2 rs3740066	2660	CC *vs*. CT + TT	Asian	R	2.53 (1.56–4.08)	0.0001	0.09	43.2	0.325	**0.143**	0.898	**0.016**	0.456	**0.545**	0.984	ABA	M
Hu 2023^47^	AEDs	GABRG2 rs211037	320	C *vs*. T	non‐Asian	F	0.23 (0.13–0.39)	(<0.0001)	<0.01	0	NC^a^	**0.023**	0.556	**0.000**	**0.024**	**0.088**	0.835	BA‐	N/A
Hu 2023^47^	AEDs	GABRG2 rs211037	160	CC *vs*. CT + TT	non‐Asian	R	0.18 (0.07–0.47)	(0.0005)	<0.01	15	NC^a^	0.701	0.992	0.322	0.962	0.891	0.998	BA‐	N/A
Hu 2023^47^	AEDs	GABRG2 rs211037	160	CC + CT *vs*. TT	non‐Asian	F	0.08 (0.02–0.40)	(0.002)	<0.01	0	NC^a^	0.890	0.998	0.757	0.994	0.937	0.999	BA‐	N/A
Mohammadi 2025^49^	AEDs	SCN1A rs2298771	15 420	G *vs*. A	Overall	F	1.20 (1.025–1.405)	0.023	(0.31)	10.64	0.186^b^	0.309	0.959	0.308	0.959	0.859	0.997	AAA	S
Mohammadi 2025^49^	AEDs	SCN1A rs2298771	7710	GG *vs*. AA+AG	Overall	F	1.35 (1.04–1.75)	0.022	(0.46)	0	0.229^b^	0.350	0.966	0.298	0.957	0.846	0.997	AAA	S
Zhang 2021^38^	CBZ	SCN1A rs2298771	941	GG *vs*. AA+GA	Asian	F	3.19 (1.27–8.02)	0.013	0.926	0	0.244	0.826	0.996	0.615	0.988	0.927	0.998	BAA	M
Mohammadi 2025^49^	AEDs	SCN1A rs10167228	478	A *vs*. G	Overall	F	1.85 (1.18–2.91)	0.007	(0.32)	0	NC^a^	0.441	0.977	**0.185**	0.923	0.838	0.996	BA‐	N/A

Abbreviations: AED, antiepileptic drug; BFDP, Bayesian false discovery probability; CBZ, carbamazepine; FPRP, false positive reporting probability; Het, heterogeneity; *I* ^2^, inconsistency; M, moderate; N/A, not applicable; NC, not calculable; OR; odds ratio; S, strong; W, weak.

^a^ Not calculable because the meta‐analysis only included two studies.

^b^ The value was provided by the corresponding author of the paper after being contacted by email.

### 3.4 eQTL analysis findings for selected SNPs

**FIGURE 2:**
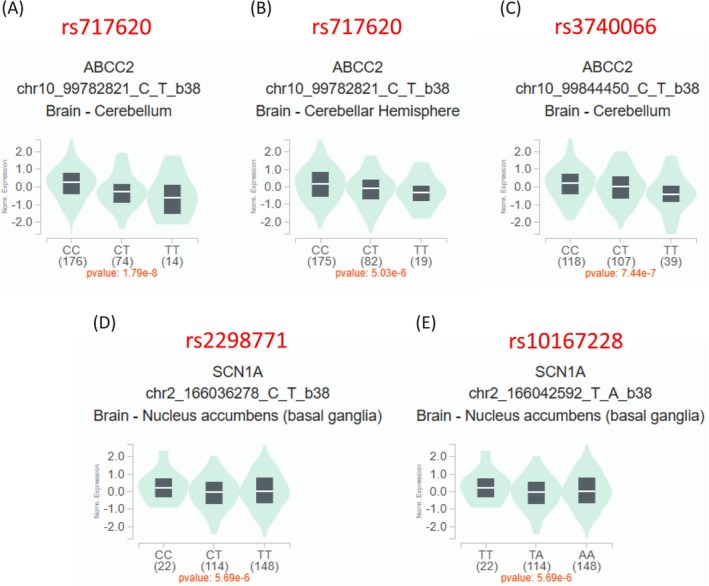
Expression quantitative trait loci (eQTL) analysis results illustrating the association between genotype and gene expression for selected SNPs in human brain tissues. The box plots illustrate the association between SNP genotype (*x*‐axis) and normalized gene expression levels (*y*‐axis) for specific gene–tissue combinations. All data were obtained from the Genotype‐Tissue Expression (GTEx) Project portal, data release v8. The panels show: (A) rs717620 genotype and ABCC2 expression in the cerebellum; (B) rs717620 genotype and ABCC2 expression in the cerebellar hemisphere; (C) rs3740066 genotype and ABCC2 expression in the cerebellum; (D) rs2298771 genotype and SCN1A expression in the nucleus accumbens (basal ganglia); (E) rs10167228 genotype and SCN1A expression in the nucleus accumbens (basal ganglia). The number of individuals per genotype is shown in parentheses. The *P*‐value below each plot indicates the statistical significance of the eQTL association, representing the effect of the alternative allele relative to the reference allele.

Publicly available eQTL datasets (e.g., GTEx Portal) were also queried to explore the putative regulatory role of selected SNPs from eligible meta‐analyses. This revealed a substantial genotype expression correlation in relevant brain tissues for the gene variants shown in Figure 2. Notably, rs717620 T allele (represented by the TT genotype) and the rs3740066 T allele (TT genotype) of ABCC2 were strongly related to a decreased ABCC2 expression in brain‐cerebellum regions (cerebellar hemisphere and cerebellum) (all *P* < 1 × 10^−5^, GTEx v8). In addition, the rs2298771 T allele (TT genotype) and the rs10167228 A allele (AA genotype) of SCN1A were significantly linked to reduced SCN1A expression in the nucleus accumbens (basal ganglia) (*P* < 1 × 10^−5^ for both, from GTEx v8). These results suggest that these specific polymorphisms have statistically significant regulatory effects on their respective gene targets in human brain tissue.

## 4 DISCUSSION

This study systematically re‐assessed the plausibility of genetic associations with antiepileptic drug (AED) response found in published meta‐analyses, through an evaluation based on methodological quality (AMSTAR‐2), epidemiological credibility (Venice criteria), and statistical robustness using Bayesian methods (FPRP, BFDP). The key finding is the paucity of strong evidence in support of the many previously reported, primarily candidate‐gene, association findings, and the concerning signal that the reliability of the existing evidence is critically low across all meta‐analyses identifieed by our systematic review. Although our re‐analysis identified seven SNPs in four candidate genes (ABCB1, ABCC2, GABRG2, SCN1A) as possible contributors to AED resistance, based on initial statistical evidence as provided in meta‐analyses, very little of these SNPs were able to withstand the joint tests on credibility and Bayesian significance evaluation.

Of the few possible signals, a putative association of ABCB1 rs2032582 (C3435T) with AED resistance was particularly complex and informative. In Caucasians, allelic (G *vs*. A) and dominant (GG *vs*. GA + AA) models were noteworthy at the stringent prior probability (0.001), indicating robustness of the obtained results. However, the Venice criteria downgraded the credibility of these specific associations to weak due to possible publication bias. Conversely, in Asian populations, this SNP (T + A *vs*. G) had a strong credibility according to Venice criteria but did not reach noteworthiness under the FPRP/BFDP. This discrepancy illustrates difficulties in interpreting genetic association studies and emphasizes the utility of using several evaluation methods. Possible reasons for this discrepancy could be true ethnic differences in genetic structure, patterns of linkage disequilibrium or environmental interaction modifying the effect of the SNP.^50^, ^51^ Alternatively, this may indicate the different sensitivities of the methods of analysis—the Venice criteria are highly sensitive to sample size and heterogeneity,^11^ and Bayesian methods are affected by assumptions about prior probabilities or adequacy of statistical power^52^, ^53^—or it could indicate enduring biases within the included studies that one or other of these approaches did not capture entirely.

To interpret our results in a broader context, we note that some of the SNPs analysed, such as rs717620 and rs3740066 (ABCC2) and rs2298771 and rs10167228 (SCN1A), act as expression quantitative trait loci (eQTLs) in relevant brain tissues, based on information available from external databases. This indicates that it has a provable biological role in that it modulates the expression of SCN1A, a crucial voltage‐gated sodium channel alpha subunit that is a frequent target for AEDs, as well as ABCC2, a major ATP‐binding cassette transporter, important in drug efflux across the blood–brain barrier.^54^, ^55^ These results generate a high degree of biological plausibility and imply potential mechanisms that mediate the impact of such genetic variants on the transport kinetics of AEDs or the neuronal excitability thresholds, ultimately affecting drug response. But this established biological significance must be very clearly separated out from a strong clinical association which is robust and replicable. Our systematic re‐evaluation underscores that even though this functional evidence stresses the relevance of these genes in epilepsy pharmacogenetics, the combined clinical evidence from existing meta‐analysis that has synthesized associations between these particular common variants and AED resistance is largely incoherent in the face of thorough investigation. The meta‐analytic methodology was of very poor quality, suffering from possible publication bias, and limitations related to the candidate gene approach probably contribute to either an underestimation or overestimation of the true associations.^56^, ^57^ Therefore, although the eQTL data support the functional relevance of these variants and support the biologic rationale of exploring these genes, they also reflect the limitation of the current clinical evidence base, mainly based on meta‐analyses of candidate genes. This underlines the necessity of robust, well‐powered genomic studies such as GWAS followed by replication to identify reliable genetic predictors of response to AEDs.

One of the most important findings of this re‐appraisal, which encompasses the identification of all the findings, is the seriously low methodological quality of the systematic reviews, which was measured with AMSTAR‐2. Such a pervasive restriction, particularly with regard to the critical issues, such as with no pre‐specified protocols (item 2), insufficient analysis of the risk of bias in the primary studies (item 9), and the risk of bias not being used as a criterion for interpretation of results (item 13), makes the reliability of the overall pooled estimates and conclusions highly questionable *ab initio*. It only further exacerbates concerns that these meta‐analyses are all limited to candidate gene studies—a paradigm that is notorious for the high probability of spurious positive findings.^56^, ^57^ A prime example of such inconsistencies is the functional intronic polymorphism rs3812718 in the SCN1A gene. This variant was initially associated with drug resistance in several studies, particularly with sodium channel‐blocking anti‐seizure drugs. However, later investigations yielded contradictory results, as many studies were unable to replicate the association. The most recent and comprehensive meta‐analysis by Mohammadi et al.,^49^ which pooled data from 25 separate studies, definitively concluded that there was no significant association between rs3812718 and drug resistance in any of the genetic models tested. This case illustrates perfectly the journey of a promising candidate gene variant from initial positive reports to eventual refutation by larger, more methodologically sound analyses. It also highlights the inherent risk of false positives in the era of gene candidates and emphasizes the value of systematic re‐evaluation. The absence of reliable associations identified in our re‐analysis is likely to largely be a result of poor quality and inherent limitations of the original evidence base.

The robust nature of this systematic review is a result of the thorough, multifaceted nature of the assessment of the evidence synthesis. In using AMSTAR‐2 for quality assessment, Venice criteria for epidemiologic grading, and Bayesian metrics (FPRP and BFDP) for statistical robustness in relation to false positives, we are able to provide a more balanced, nuanced set of inferences about the available evidence than did the original publications. The framework extends far beyond the crude applications of *P*‐value thresholds, and when used correctly it can help ensure that statistically significant findings are in fact true positives. However, this re‐assessment has limitations despite methodological rigour. The conclusions of this overview are limited to the quality and quantity of the underlying evidence covered in the included meta‐analyses. We were only able to evaluate the data as written, and the inherent flaws in the original studies (e.g., disparities in phenotype definitions, insufficient control for confounding factors, misclassification due to genotyping error) cannot be remedied in retrospect. Some stress that by specifically re‐evaluating meta‐analyses we did not include results of GWASs; this is a deliberate focus, and the need for such studies is a clear answer. Lastly, the context heterogeneity (e.g., AEDs used, patient populations, definitions of response/resistance) and follow‐up duration of the studies analysed in the original meta‐analyses also probably contributes to the inconsistencies observed and limits the generalizability of the potential results.

The genes related to the statistically significant but largely non‐credible associations (ABCB1, ABCC2, SCN1A, GABRG2) are plausible biologic candidates for the transport of AEDs across the blood–brain barrier (ABCB1, ABCC2) or for neuronal excitability (SCN1A, GABRG2)^54^, ^55^, ^58^ as also supported by the eQTL findings as described. Nevertheless, the weak associations reported here are in line with the generally underwhelming results of candidate gene studies for complex traits. This conclusion is further substantiated by GWAS, which employ a hypothesis‐free approach. For example, Wolking et al. conducted a GWAS to assess the role of common variants in response to certain AEDs and found no genome‐wide significant associations, suggesting that there are no variants with large effect on individual drug response.^59^ More recently, the largest GWAS meta‐analysis to date on drug resistance by Leu et al. also found no significant loci when they analysed more than 6800 individuals with different types of epilepsy together.^60^ This failure of the data‐driven, hypothesis‐agnostic approach of GWAS to identify broadly applicable predictors is largely consistent with the conclusions of our own re‐evaluation of meta‐analyses with candidate genes. Remarkably, the study by Leu et al. actually identifies a significant locus associated with drug response, but only when the analysis was restricted to the large subgroup of patients with focal epilepsy.^60^ This highlights that while common gene variants may indeed contribute to drug resistance, their effects are likely to be modest and specific to certain epilepsy subtypes and require very large, well‐phenotyped cohorts to be reliably detected. This contrasts with the claims of the many inadequate candidate gene studies we have reviewed and emphasizes our call for large‐scale, methodologically rigorous genomic studies to identify robust predictors of resistance to AEDs.

Furthermore, it is important to recognize the paradigm shift in epilepsy genetics towards the study of rare and extremely rare variants. The advent of exome and genome sequencing has shown that high‐impact rare variants, including single nucleotide variants (SNVs) and copy number variations (CNVs), are critical contributors to the aetiology of drug‐resistant epilepsy (DRE), particularly in severe developmental and epileptic encephalopathies (DEEs).^61^ For example, loss‐of‐function variants in SCN1A are strongly associated with Dravet syndrome, a condition characterized by marked drug resistance.^62^, ^63^ This line of research is not limited to severe early‐onset syndromes. Recent findings suggests that enrichment of rare variants may also contribute to DRE in other contexts, such as non‐lesional focal epilepsy. For instance, a study by Wolking et al. found an enrichment of rare truncating variants in known focal epilepsy genes (e.g., DEPDC5) in non‐responders to AEDs.^64^ This shows that the genetic architecture of drug resistance is very heterogeneous and covers a spectrum from common variants with minor effects to rare variants with large, deterministic effects. While common variants have so far shown limited predictive power, the study of rare variants offers a promising avenue for understanding the multifactorial nature of treatment resistance and for identifying patients who could benefit from precision medicine strategies. The clinical relevance of our results is straightforward: according to the current assessment of the recently rigorously re‐evaluated evidence from those meta‐analyses of mainly candidate gene studies, there is no clinical reason for the use of the tested polymorphisms (rs1045642, rs2032582 in ABCB1; rs717620, rs3740066 in ABCC2; rs211037 in GABRG2; rs2298771, rs10167228 in SCN1A) in guiding clinical decisions about drug selection or dosing of AEDs for the risk prediction of drug resistance. The genetic testing indicated by this evidence is premature and likely to be misleading.

The recognized absence of valid genetic markers necessitates a shift of approach to search for reliable predictors of AED responsiveness. It will take a bold leap towards broad genome approaches targeting pharmacogenomic phenotypes to get beyond the current constraints. Success relies on large numbers and precise, and standardized, phenotype definitions. A rigorous independent validation study in multiple cohorts is essential before clinical application. Uniform definitions of all patient‐related clinical outcomes (e.g., resistance to AEDs) are essential at every stage in order to minimize heterogeneity and allow meaningful comparisons. Furthermore, study design for better progress needs to be more complicated. These include, but are not limited to, introduction of prospective elements where possible, conducting drug‐specific analyses both singly and in categories of similarly acting AEDs, stratification of analyses according to relevant variables such as ethnicity, age and epilepsy syndrome, and exploration and testing of potential gene–gene and gene–environment interactions. Finally, such improved primary evidence also needs to be better synthesized. Future systematic reviews and meta‐analyses should rely on high‐quality primary studies, especially good design of GWAS and their replications, and they have to apply their own rigorous methodological standards, including registration of protocols, careful consideration of risk of bias, use of sensible statistical techniques, possibly also applying Bayesian methods and they should make transparent how and why they have adjusted for any remaining bias and heterogeneity in their interpretation. The utility of such tools as trial sequential analysis (TSA) could assist in determining when the data has been sufficient to draw finite conclusions.

## 5 CONCLUSION

In summary, this systematic Bayesian review of meta‐analyses reveals a resounding absence of credible evidence for the associations derived from all available evidence of common genetic polymorphisms assessed via the application of the candidate gene approach in determining AED resistance. The critically low quality of current meta‐analyses also undermines the evidence base on this topic. The field now needs to transition from small, underpowered candidate gene studies and low‐quality evidence syntheses, and instead pursue large unbiased genomic studies combined with rigorous methodological replication and meta‐analyses, to finally uncover reliable genetic predictors that one day may lead to personalized epilepsy treatment.

### 5.1 Nomenclature of targets and ligands

Key protein targets and ligands in this article are hyperlinked to corresponding entries in http://www.guidetopharmacology.org, and are permanently archived in the Concise Guide to PHARMACOLOGY 2023/24.^65^, ^66^

## AUTHOR CONTRIBUTIONS

S.T. coordinated the study design, data acquisition, and drafted the initial manuscript. All authors participated in data analysis and interpretation. M.G. and S.C. provided critical review and revisions to the manuscript. All authors read and approved the final manuscript. The corresponding author (S.T.) attests that all listed authors meet the criteria for authorship.

## CONFLICT OF INTEREST STATEMENT

All authors reported no conflicts of interest.

## Supporting information


**Table S1.** Summary of non‐significant results from meta‐analyses investigating associations between genetic variants and response to anti‐seizure medications.

## Data Availability

The data presented in this study are available in the main text and supplementary materials associated with this article.
