# Supplementary material for: Credibility of genetic predictors for antiepileptic drug resistance: A systematic Bayesian reappraisal of published meta‐analyses
Source: Br J Clin Pharmacol. 2025 Aug 1;91(10):2782–95. doi: 10.1002/bcp.70189 (PMC12464618; doi:10.1002/bcp.70189)
Supplement: Supplementary file 1 — Table S1. Summary of non‐significant results from meta‐analyses investigating associations between genetic variants and response to anti‐seizure medications. [file BCP-91-2782-s001.pdf]

Table S1. Summary of non-significant results from meta-analyses investigating associations between genetic variants and response to anti-seizure medications[a].

Study[Ref.]	*Antiepileptic Drug*	*Gene variant*	*Comparison*	*Total subjects or alleles*	*Ethnicity*	*Model*	*OR (95% CI)*	*P-value*	P_{het}	I^2 *(%)*	*Egger (P-value)*
Li 2015[31]	AEDs	ABCB1 rs1128503	T vs C	NR	Overall	F	0.97 (0.90-1.06)	0.56	0.51	NR	0.218
Li 2015[31]	AEDs	ABCB1 rs1128503	TT vs TC+CC	NR	Overall	F	0.97 (0.86-1.09)	0.63	0.65	NR	NR
Li 2015[31]	AEDs	ABCB1 rs1128503	TT+TC vs CC	NR	Overall	F	0.99 (0.84-1.16)	0.88	0.75	NR	NR
Li 2015[31]	AEDs	ABCB1 rs1128503	T vs C	NR	Caucasian	F	0.82 (0.66-1.00)	0.06	0.24	NR	NR
Li 2015[31]	AEDs	ABCB1 rs1128503	TT vs TC+CC	NR	Caucasian	F	0.73 (0.51-1.04)	0.08	0.53	NR	NR
Li 2015[31]	AEDs	ABCB1 rs1128503	TT+TC vs CC	NR	Caucasian	F	0.84 (0.57-1.25)	0.40	0.13	NR	NR
Li 2015[31]	AEDs	ABCB1 rs1128503	T vs C	NR	Asian	F	1.04 (0.93-1.16)	0.47	0.54	NR	NR
Li 2015[31]	AEDs	ABCB1 rs1128503	TT vs TC+CC	NR	Asian	F	1.07 (0.92-1.25)	0.36	0.64	NR	NR
Li 2015[31]	AEDs	ABCB1 rs1128503	T vs C	NR	Indian	F	0.94 (0.81-1.11)	0.48	0.82	NR	NR
Li 2015[31]	AEDs	ABCB1 rs1128503	TT vs TC+CC	NR	Indian	F	0.88 (0.70-1.10)	0.26	0.85	NR	NR
Li 2015[31]	AEDs	ABCB1 rs1128503	TT+TC vs CC	NR	Indian	F	1.03 (0.77-1.38)	0.86	0.73	NR	NR
Li 2015[31]	AEDs	ABCB1 rs1128503	T vs C	NR	Adult	F	0.95 (0.81-1.11)	0.54	0.10	NR	NR
Li 2015[31]	AEDs	ABCB1 rs1128503	TT vs TC+CC	NR	Adult	F	0.99 (0.78-1.26)	0.94	0.25	NR	NR
Li 2015[31]	AEDs	ABCB1 rs1128503	TT+TC vs CC	NR	Adult	F	0.92 (0.66-1.27)	0.61	0.21	NR	NR
Li 2015[31]	AEDs	ABCB1 rs1128503	T vs C	NR	Children	F	0.91 (0.76-1.09)	0.31	0.54	NR	NR
Li 2015[31]	AEDs	ABCB1 rs1128503	TT vs TC+CC	NR	Children	F	0.91 (0.70-1.18)	0.49	0.72	NR	NR
Li 2015[31]	AEDs	ABCB1 rs1128503	TT+TC vs CC	NR	Children	F	0.86 (0.63-1.19)	0.37	0.44	NR	NR
Fan 2021[39]	CBZ	ABCB1 rs1128503	T vs C	NR	Mixed	R	0.68 (0.25-1.83)	0.45	NR	80	NR
Fan 2021[39]	CBZ	ABCB1 rs1128503	TT vs TC+CC	NR	Mixed	R	0.70 (0.23-2.16)	0.54	NR	72	NR
Fan 2021[39]	CBZ	ABCB1 rs1128503	TT+TC vs CC	NR	Mixed	R	0.36 (0.06-2.06)	0.25	NR	65	NR
Zan 2021[40]	AEDs	ABCC2 rs2273697	AA vs GA+GG	4862	Overall	F	1.12 (0.97-1.28)	0.10	0.19	NR	NR
Zan 2021[40]	AEDs	ABCC2 rs2273697	AA+GA vs GG	4862	Overall	F	1.01 (0.68-1.49)	0.96	0.19	NR	NR
Zan 2021[40]	AEDs	ABCC2 rs2273697	AA vs GA+GG	4163	Asian	F	1.12 (0.97-1.30)	0.14	0.10	NR	NR
Zan 2021[40]	AEDs	ABCC2 rs2273697	AA+GA vs GG	4163	Asian	F	1.26 (0.78-2.03)	0.34	0.42	NR	NR
Zan 2021[40]	AEDs	ABCC2 rs2273697	AA vs GA+GG	699	Caucasian	F	0.64 (0.32-1.27)	0.20	0.11	NR	NR

Study[Ref.]	*Antiepileptic Drug*	*Gene variant*	*Comparison*	*Total subjects or alleles*	*Ethnicity*	*Model*	*OR (95% CI)*	*P-value*	P_{het}	I^2 *(%)*	*Egger (P-value)*
Zan 2021[40]	AEDs	ABCC2 rs2273697	AA+GA vs GG	699	Caucasian	F	1.10 (0.76-1.59)	0.61	0.70	NR	NR
Wang 2015[28]	AEDs	ABCC2 rs3740070	G vs A	628	Asian	F	1.09 (0.42-2.83)	0.85	0.98	0	NR
Wang 2015[28]	AEDs	ABCC2 rs3740070	GG+GA vs AA	314	Asian	NR	NR	NR	NR	NR	NR
Wang 2015[28]	AEDs	ABCC2 rs3740070	GG vs GA+AA	314	Asian	NR	NR	NR	NR	NR	NR
Zan 2021[40]	AEDs	ABCG2 rs2231137	TT vs CT+CC	NR	Overall	F	1.07 (0.84-1.37)	0.59	0.99	NR	NR
Zan 2021[40]	AEDs	ABCG2 rs2231137	CT+TT vs CC	NR	Overall	F	1.03 (0.71-1.49)	0.87	0.31	NR	NR
Wang 2015[28]	AEDs	ABCC2 −1774G>delG	G vs delG	1186	Asian	R	1.18 (0.64-2.17)	0.60	0.01	79	NR
Wang 2015[28]	AEDs	ABCC2 −1774G>delG	GG vs GdelG+delGdelG	592	Asian	R	1.04 (0.47-2.32)	0.92	0.01	79	NR
Zan 2021[40]	AEDs	ABCG2 rs2231142	GC+CC vs CC	NR	Overall	F	1.11 (0.87-1.41)	0.39	0.72	NR	NR
Zan 2021[40]	AEDs	ABCG2 rs2231142	CC vs CT+CC	NR	Overall	F	0.88 (0.58-1.33)	0.54	0.82	NR	NR
Zhao 2019[37]	CBZ	EPHX1 rs1051740	CC vs CT+TT	88	Overall	F	1.10 (0.66-1.83)	0.71	0.62	0	NR
Zhao 2019[37]	CBZ	EPHX1 rs1051740	CC+CT vs TT	305	Overall	F	0.89 (0.60-1.34)	0.59	0.18	39	NR
Zhao 2019[37]	CBZ	EPHX1 rs2234922	GG vs GA+AA	20	Overall	F	1.43 (0.58-3.52)	0.44	0.77	0	NR
Zhao 2019[37]	CBZ	EPHX1 rs2234922	GG+GA vs AA	141	Overall	F	0.97 (0.62-1.53)	0.90	0.76	0	NR
Zhang 2022[45]	AEDs	GABRA1 rs2279020	GG vs GA+AA	NR	Overall	F	1.17 (0.94-1.45)	0.15	0.18	NR	NR
Zhang 2022[45]	AEDs	GABRA1 rs2279020	GA+GG vs AA	NR	Overall	R	1.21 (0.82-1.77)	0.33	0.01	71.1	NR
Zhang 2022[45]	AEDs	GABRA1 rs2279020	GA+GG vs AA	NR	Asian	F	1.18 (0.94-1.48)	0.27	0.11	NR	NR
Zhang 2022[45]	AEDs	GABRA1 rs2279020	GG vs GA+AA	NR	Asian	R	1.09 (0.73-1.63)	0.67	0.02	NR	NR
Zhang 2022[45]	AEDs	GABRA6 rs3219151	TT vs CT+CC	NR	Overall	F	1.26 (0.98-1.63)	0.08	0.29	NR	NR
Zhang 2022[45]	AEDs	GABRA6 rs3219151	CC vs CT+TT	NR	Overall	F	0.95 (0.71-1.27)	0.73	0.93	NR	NR
Mohammedi 2025[49]	AEDs	SCN1A rs10188577	Allelic	10962	Overall	F	0.99 (0.90-1.09)	0.80	NR	43.49	NR
Mohammedi 2025[49]	AEDs	SCN1A rs10188577	Recessive	5481	Overall	F	0.82 (0.62-1.08)	0.16	NR	47.15	NR
Mohammedi 2025[49]	AEDs	SCN1A rs1020853	Allelic	1966	Overall	F	0.93 (0.76-1.13)	0.46	NR	0	NR
Mohammedi 2025[49]	AEDs	SCN1A rs1020853	Recessive	983	Overall	F	1.04 (0.70-1.55)	0.84	NR	26.08	NR
Mohammedi 2025[49]	AEDs	SCN1A rs1461197	Allelic	1782	Overall	F	0.86 (0.70-1.06)	0.17	NR	0	NR
Mohammedi 2025[49]	AEDs	SCN1A rs1461197	Recessive	891	Overall	F	0.78 (0.50-1.23)	0.29	NR	0	NR

Study[Ref.]	*Antiepileptic Drug*	*Gene variant*	*Comparison*	*Total subjects or alleles*	*Ethnicity*	*Model*	*OR (95% CI)*	*P-value*	P_{het}	I^2 *(%)*	*Egger (P-value)*
Bao 2018[35]	SCB-AEDs	SCN1A rs3812718	A vs G	4204	Overall	F	0.98 (0.86-1.11)	0.73	0.77	0	0.33
Bao 2018[35]	SCB-AEDs	SCN1A rs3812718	AA vs AG+GG	2102	Overall	F	0.99 (0.81-1.20)	0.92	0.99	0	0.56
Bao 2018[35]	SCB-AEDs	SCN1A rs3812718	AA+AG vs GG	2102	Overall	F	0.95 (0.77-1.17)	0.64	0.35	10	0.23
Bao 2018[35]	SCB-AEDs	SCN1A rs3812718	A vs G	1634	Chinese	F	0.89 (0.73-1.08)	0.23	0.44	0	NR
Bao 2018[35]	SCB-AEDs	SCN1A rs3812718	AA vs AG+GG	817	Chinese	F	0.91 (0.67-1.22)	0.52	0.95	0	NR
Bao 2018[35]	SCB-AEDs	SCN1A rs3812718	AA+AG vs GG	817	Chinese	F	0.80 (0.57-1.11)	0.18	0.27	24	NR
Wang 2018[36]	VPA	SCN1A rs3812718	TT vs TC+CC	4951	Overall	R	0.86 (0.70-1.06)	0.15	0.02	48.7	NR
Wang 2018[36]	VPA	SCN1A rs3812718	TT vs TC+CC	1372	Caucasian	R	0.99 (0.76-1.27)	0.91	0.48	0	NR
Wang 2018[36]	VPA	SCN1A rs3812718	TT vs TC+CC	3579	Asian	R	0.83 (0.64-1.08)	0.16	0.01	56.2	NR
Zhang 2021[38]	CBZ	SCN1A rs3812718	A vs G	2220	Overall	F	1.25 (0.83-1.89)	0.29	0.88	71	0.97
Zhang 2021[38]	CBZ	SCN1A rs3812718	AA vs GA+GG	1110	Overall	F	1.50 (0.94-2.40)	0.09	0.79	60.9	0.88
Zhang 2021[38]	CBZ	SCN1A rs3812718	AA+GA vs GG	1110	Overall	F	1.04 (0.76-1.43)	0.33	0.64	13.7	0.80
Zhang 2021[38]	CBZ	SCN1A rs3812718	A vs G	NR	European	F	0.95 (0.67-1.33)	0.75	0.83	0	NR
Zhang 2021[38]	CBZ	SCN1A rs3812718	AA vs GA+GG	NR	European	F	1.14 (0.62-2.09)	0.68	0.75	0	NR
Zhang 2021[38]	CBZ	SCN1A rs3812718	AA+GA vs GG	NR	European	F	0.78 (0.45-1.35)	0.38	0.36	0	NR
Zhang 2021[38]	CBZ	SCN1A rs3812718	A vs G	NR	Asian	R	1.51 (0.79-2.87)	0.21	0.01	78.6	0.95
Zhang 2021[38]	CBZ	SCN1A rs3812718	AA vs GA+GG	NR	Asian	R	1.70 (0.88-3.25)	0.11	0.01	74.7	0.64
Zhang 2021[38]	CBZ	SCN1A rs3812718	AA+GA vs GG	NR	Asian	F	1.21 (0.82-1.79)	0.34	0.33	9.9	0.55
Haerian 2013[21]	AEDs	SCN1A rs3812718	A vs G	4950	Overall	F	0.94 (0.85-1.05)	0.39	0.39	6	NR
Haerian 2013[21]	AEDs	SCN1A rs3812718	AA vs AG+GG	2475	Overall	F	0.87 (0.74-1.02)	0.09	0.16	0	NR
Mohammedi 2025[49]	AEDs	SCN1A rs1972445	Allelic	1182	Overall	R	0.83 (0.51–1.35)	0.46	NR	0	NR
Mohammedi 2025[49]	AEDs	SCN1A rs1972445	Recessive	591	Overall	R	0.83 (0.54–1.28)	0.41	NR	0	NR
Mohammedi 2025[49]	AEDs	SCN1A rs3812718	Allelic	13128	Overall	F	1.02 (0.95–1.09)	0.63	NR	49.93	NR
Mohammedi 2025[49]	AEDs	SCN1A rs3812718	Recessive	6443	Overall	F	0.97 (0.86–1.11)	0.69	NR	22.86	NR
Yang 2021[41]	AEDs	SCN2A rs17183814	G vs A	6058	Asian	F	0.97 (0.83-1.20)	0.78	0.13	NR	NR
Yang 2021[41]	AEDs	SCN2A rs17183814	GG vs GA+AA	3029	Asian	F	0.95 (0.81-1.13)	0.56	0.54	NR	NR

Study[Ref.]	Antiepileptic Drug	Gene variant	Comparison	Total subjects or alleles	Ethnicity	Model	OR (95% CI)	P-value	P_{het}	I^2 (%)	Egger (P-value)
Yang 2021[41]	AEDs	SCN2A rs17183814	GG+GA vs AA	3029	Asian	F	1.03 (0.64-1.65)	0.90	0.12	NR	NR
Yang 2021[41]	AEDs	SCN2A rs2304016	A vs G	2876	Asian	R	0.88 (0.56-1.39)	0.58	0.03	NR	NR
Yang 2021[41]	AEDs	SCN2A rs2304016	AA vs AG+GG	1438	Asian	R	1.18 (0.66-2.10)	0.57	0.01	NR	NR
Yang 2021[41]	AEDs	SCN2A rs2304016	AA+AG vs GG	1438	Asian	F	0.73 (0.26-2.02)	0.54	0.81	NR	NR
Hu 2023[47]	AEDs	SLC6A11 rs2304725	T vs C	5010	Mixed	F	0.96 (0.85-1.09)	NR	0.32	15	0.59
Hu 2023[47]	AEDs	SLC6A11 rs2304725	TT vs TC+CC	2505	Mixed	F	0.99 (0.83-1.17)	NR	0.51	0	0.47
Hu 2023[47]	AEDs	SLC6A11 rs2304725	TT+TC vs CC	2505	Mixed	F	0.89 (0.74-1.08)	NR	0.70	0	0.50
Haerian 2011[20]	AEDs	haplotype rs1045642/rs2032582/rs1128503	CGC vs TTT	NR	Overall	NR	NR	NR	NR	NR	NR
Li 2015[31]	AEDs	haplotype rs1128503/rs1045642/rs2032582	TTT vs non-TTT	2567	Overall	R	1.31 (0.94-1.81)	0.11	<0.01	NR	NR
Li 2015[31]	AEDs	haplotype rs1128503/rs1045642/rs2032582	TTT vs CGC	2567	Overall	R	1.04 (0.82-1.32)	0.72	0.04	NR	NR
Li 2015[31]	AEDs	haplotype rs1128503/rs1045642/rs2032582	Non-CGC vs CGC	2567	Overall	R	0.83 (0.51-1.35)	0.46	<0.01	NR	NR
Li 2015[31]	AEDs	haplotype rs1128503/rs1045642/rs2032582	TTT vs non-TTT	NR	Asian	R	1.45 (0.83-2.52)	0.19	<0.01	NR	NR
Li 2015[31]	AEDs	haplotype rs1128503/rs1045642/rs2032582	TTT vs CGC	NR	Asian	F	0.99 (0.74-1.33)	0.96	0.13	NR	NR
Li 2015[31]	AEDs	haplotype rs1128503/rs1045642/rs2032582	Non-CGC vs CGC	NR	Asian	R	0.69 (0.32-1.48)	0.34	<0.01	NR	NR
Li 2015[31]	AEDs	haplotype rs1128503/rs1045642/rs2032582	TTT vs non-TTT	NR	Indian	F	1.10 (0.90-1.34)	0.35	0.52	NR	NR
Li 2015[31]	AEDs	haplotype rs1128503/rs1045642/rs2032582	TTT vs CGC	NR	Indian	R	1.15 (0.73-1.83)	0.53	0.03	NR	NR
Li 2015[31]	AEDs	haplotype rs1128503/rs1045642/rs2032582	Non-CGC vs CGC	NR	Indian	R	1.08 (0.68-1.71)	0.74	0.01	NR	NR
Qian 2017[32]	AEDs	haplotype rs717620/rs2273697/rs3740066	CAC vs non-CAC	NR	Overall	R	1.05 (0.73–1.50)	0.81	0.08	55	NR
Qian 2017[32]	AEDs	haplotype rs717620/rs2273697/rs3740066	TGT vs non–TGT	NR	Overall	F	1.23 (0.99–1.53)	0.06	0.14	45	NR
Qian 2017[32]	AEDs	haplotype rs717620/rs2273697/rs3740066	CGC vs non-CGC	NR	Overall	R	1.07 (0.81–1.41)	0.63	0.06	60	NR
Wang 2015[28]	AEDs	haplotype−24C-1249G-3972C vs -24T-1249A-3972T	CGC vs TAT	1152	Asian	R	0.87 (0.55-1.38)	0.56	0.05	49	0.39

[a]For genetic polymorphisms included in multiple meta-analyses, results from the meta-analysis with the largest sample size are presented, unless the results regard different subgroups (eg, ethnic group, antiepileptic drug type, or pediatric status). AED, antiepileptic drug; CBZ, carbamazepine; CI, confidence interval; F, fixed-effects model; I^2, inconsistency; NR, not reported; OR, odds ratio; R, random-effects model; Ref., reference; SCB-AEDs, sodium channel blocking antiepileptic drugs; VPA, valproic acid.
